# Discretionary Bequests

**Published:** 1896-02-15

**Authors:** 


### The Institutional Workshop.

### DISCRETIONARY BEQUESTS.

### IMPORTANT CONSIDERATIONS FOR HOSPITALS AND CHARITIES.

H. H. G. writes: In common with other hospital secretaries I receive, from time to time, intelligence of wills by which the testator leaves property (generally "residue") in trust to his executors to "distribute in their absolute and uncontrolled discretion to such charities or charitable societies as they may select." In some such words as these many thousands of pounds are bequeathed each year in this country, and I and other secretaries do not fail to make application for a share in the distribution.

Then, with more or less hope, we await the result. Sometimes we are favoured, sometimes "left out in the cold." But not infrequently we never hear that any distribution has taken place, or that any institution has been benefited. Now this complete ignorance not unnaturally arouses uncomfortable suspicions as to whether the money has been, or ever will be, distributed in accordance with the terms of the trust; and, human nature being what it is, it does not imply a very cynical or suspicious turn of mind if one ventures to wonder whether, in some cases, the money may not have been diverted to other objects more in accordance with the maxim that "charity begins at home," and may have even reached the pockets of the next of kin.

I at once plead guilty to such wonderings, and in the spring of last year, having made it my business to consult some other hospital secretaries, I found that they also were base enough to entertain the suspicions I have indicated.

The question then presented itself to my mind: Is it anybody's business (a) to inquire whether these trusts have been carried out; (b) to enforce their performance?

My brother secretaries, aforesaid, promised to gain information in these points, if possible; but I heard nothing from them on the matter, and I therefore determined to take it up myself.

Assisted by an introduction you were good enough to give me, and by the kindness of legal friends, I was enabled to consult some eight barristers and solicitors as to the two points raised above. Without trespassing too far on your space, I may say that not one of my advisers was able to point with certainty to any recognised authority upon whom the duties indicated above appeared to devolve. The Legacy Department, Somerset House, the Attorney-General, and the Charity Commissioners were, however, referred to as possibly competent to act, and to those quarters I therefore directed my inquiries. At Somerset House I found, as I expected, that they neither possessed nor could they obtain any information. As long as the proper duty of 10 per cent. was paid they did not concern themselves as to the actual receipt of the distribution. With the solicitor to the Attorney-General I had an interview, which his helpfulness and courtesy rendered most satisfactory, though all he could tell me was that in any case where the Attorney-General had before him evidence pointing to a possible diversion of a trust of this character, he would intervene, but that he would not, in all probability, consider it his duty to make inquiry in all cases. The solicitor, however, entirely agreed with me and the other lawyers whom I had consulted, that there was an apparent need of systematic inquiry by some duly constituted authority; but, like myself and like my legal friends, he could only suggest legislation. This, however, he thought might be more easily obtained by a short clause in a Bill emanating from the Charity Commission, should that body have a suitable Parliamentary measure in view, than by an independent measure. A friend of mine who at one time held office in the Commission had kindly given me a letter to the secretary, to whom in due course I presented myself (and the letter), and put the whole matter before him.

Here, again, I met with the greatest courtesy and consideration, but with a very different outcome, for I found that the secretary was clearly of opinion that his department not only had the power to do all I had hoped for, but that it was their duty to do it; and that in point of fact, in certain cases, of which examples were readily given me, the Department had taken steps of this nature.

To carry out, however, a systematic plan of inquiry and action in all cases would need the organisation of certain details not yet within the reach of the Department.

Having thus unofficially discussed the matter, I wrote to the secretary a letter putting to him certain questions, and I now subjoin his reply to the same:—

Charity Commission, April 6th, 1895.

Sir,—Your communication of the 18th ult. has been under consideration, and I am to enclose, for your information, a Statement containing the Questions raised by you with answers thereto, arranged for the sake of convenience in categorical form.

I may add that instructions have been given that steps should be taken for obtaining the necessary particulars in those cases which have now been brought for the first time to the notice of the Commissioners, and for the completion of the information already in the possession of the Commissioners in the other cases.—I am, Sir, your obedient servant,

(Signed) D. R. Fearon.

1. Can you inform me whether the amounts referred to have been distributed by the Executors in accordance with the terms of the Wills?—The Commissioners have information in a few cases only, which information is in course of completion where necessary.

2. If the distributions have been so carried out, is your Department in possession of lists of the Charities in favour of which the distributions have been effected?—See answer to No. 1.

3. If you are able to give an affirmative reply to questions 1 and 2, on what evidence is your reply based? *e.g.*, on affidavits made by the executors?—On the statements of the Executors, to be confirmed, if necessary, by the Institutions benefited.

4. If in any case or cases you are not in a position to reply in the affirmative to questions 1, 2, and 3, is it within the province or duties of your department to ascertain by inquiry from the Executors, or otherwise, whether they have carried out the trusts imposed upon them?—Yes.

5. And if so, will your Department undertake to make such inquiries in all or any of the cases detailed in schedule A?—Yes.

6. Would it be within the duties or province of your department to take steps to enforce distribution according to the terms of the Wills?—Yes, if the action of the Commissioners should be invoked or otherwise required.

(a) If the Executors refuse or neglect to supply your department with the information indicated?—Yes.

(b) If the Executors, not having carried out the trusts imposed upon them, decline or neglect to carry out the distributions?—Yes.

The schedule referred to in my letter contained particulars of all discretionary bequests during the last nine years, excluding those cases in which I had some evidence of distribution having taken place, and when I found that the total was about half-a-million I felt it was worth looking after. At the risk of trespassing still further upon your space I will conclude by saying that I have delayed the publication of these particulars till I have been able to ascertain that the necessary machinery is in full working order at the Department in question; that the initial difficulties (greater than might be supposed) in obtaining the preliminary particulars necessary in each case have been surmounted; that inquiries have now been and are still being made in all these cases of discretionary bequests; and lastly, that it is readily recognised in the Department that the system I have been fortunate enough to set in motion has been amply justified by its results. The importance of the matter must be my excuse for the length of this letter.

